# Robotic liver parenchymal transection using the SynchroSeal

**DOI:** 10.1007/s00464-024-11005-4

**Published:** 2024-07-08

**Authors:** Gabriela Pilz da Cunha, Celine De Meyere, Mathieu D’Hondt, Rutger-Jan Swijnenburg

**Affiliations:** 1 Department of Surgery, Amsterdam UMC, Location University of Amsterdam, Amsterdam, The Netherlands; 2 Cancer Center Amsterdam, Amsterdam, The Netherlands; 3 Department of Digestive and Hepatobiliary/Pancreatic Surgery, Groeninge Hospital, Kortrijk, Belgium

**Keywords:** SynchroSeal, Energy device, Robotic liver surgery, Minimally invasive liver surgery, Parenchymal transection

## Abstract

**Background:**

There is much heterogeneity in the instrumentation used for parenchymal transection in minimally invasive liver surgery. Instruments specifically designed for robotic parenchymal transection of the liver are lacking. We aim to gain insight into the safety and effectiveness of the SynchroSeal (Intuitive Surgical, Inc., Sunnyvale, CA), a novel bipolar electrosurgical device, in the context of liver surgery.

**Methods:**

The present study is a *post-hoc* analysis of prospectively collected data from patients undergoing robotic liver resection (RLR) using the SynchroSeal in two high-volume centres. The results of the SynchroSeal were compared with that of the previous generation bipolar-sealer; Vessel Sealer Extend (Intuitive Surgical, Inc., Sunnyvale, CA) using propensity score matching, after excluding the first 25 Vessel Sealer procedures per center.

**Results:**

During the study period (February 2020–March 2023), 155 RLRs meeting the eligibility criteria were performed with the SynchroSeal (after implementation in June 2021) and 145 RLRs with the Vessel Sealer. Excellent outcomes were achieved when performing parenchymal transection with the SynchroSeal; low conversion rate (*n* = 1, 0.6%), small amounts of intraoperative blood loss (median 40 mL [IQR 10–100]), short hospital stays (median 3 days [IQR 2–4]), and adequate overall morbidity (19.4%) as well as severe morbidity (11.0%). In a matched comparison (*n* = 94 vs *n* = 94), the SynchroSeal was associated with less intraoperative blood loss (48 mL [IQR 10–143] vs 95 mL [IQR 30–200], p = 0.032) compared to the Vessel Sealer. Other perioperative outcomes were similar between the devices.

**Conclusion:**

The SynchroSeal is a safe and effective device for robotic liver parenchymal transection.

The parenchymal transection phase in liver surgery is a highly complex stage of the operation. The unique anatomy of the liver, with its dense composition of vasculature and biliary ducts makes its surgical manipulation especially vulnerable to serious complications such as bleeding and bile leakage. Transection of the parenchyma requires specialized instruments which can dissect, seal, cut, and coagulate tissue. Increased blood loss during liver surgery strongly correlates with unfavourable patient outcomes [1–3]. Hence, the ability to transect the liver parenchyma carefully and precisely is essential. Various techniques and surgical instruments have been developed for parenchymal transection during liver resection. Reported techniques include finger-fracture, clamp-crush, monopolar, bipolar, water-jet dissection, radiofrequency dissection, stapling and ultrasonic dissection [4, 5]. No consensus exists with regards to an optimal technique.

The robotic platform allows surgeons to operate with similar freedom of movement as in open surgery, with the clinical benefits of minimally invasive procedures such as accelerated recovery [6]. The wrist-like range of motion and enhanced three dimensional imaging inherent to robotic surgery gives surgeons added control which could potentially facilitate parenchymal transection. A systematic review revealed reduced intraoperative blood loss in robotic liver surgery compared with laparoscopy [7].

While instruments like the ultrasonic dissector gained much popularity in laparoscopic and open liver surgery, the device could not be directly translated to the robotic platform because of the inability to integrate it with the articulating wrist function. The introduction of the robotic console in liver surgery required medical technology companies to develop new instruments. Presently, devices used in fully-robotic liver resection are Harmonic Ace + 7 (Intuitive Surgical, Inc., Sunnyvale, CA), Vessel Sealer Extend (Intuitive Surgical, Inc., Sunnyvale, CA) and robotic bipolar graspers (Intuitive Surgical, Inc., Sunnyvale, CA) [8, 9]. A novel tool has recently been brought onto the market, namely the SynchroSeal (Intuitive Surgical, Inc., Sunnyvale, CA), designed to overcome some of the drawbacks of the aforementioned tools. The SynchroSeal is an articulating radiofrequency seal-and-transection device. It has shown promising results in ex- and in-vivo porcine models; with greater slip resistance force and faster sealing and cooling times and enhanced dexterity compared with the straight Harmonic Ace +7 [10].

There are currently no published reports on the use of the SynchroSeal for liver parenchymal transection. To date, experience with the device has been limitedly described in the context of bariatric procedures, oesophagectomy and adrenalectomy [11, 12]. The aim of the present study is to analyse the safety and effectiveness of the SynchroSeal for robotic liver parenchymal transection.

## Methods

### Study design

This is a retrospective cohort study of robotic liver resection (RLR) using the SynchroSeal energy device performed at two high volume hepatobiliary centres. All consecutive patients undergoing robotic liver resection at the Amsterdam UMC and Groeninge Hospital in Kortrijk who underwent robotic liver resection whereby the SynchroSeal or Vessel Sealer device were utilized during parenchymal transection were included. Liver transplant hepatectomies, cyst fenestrations, biopsies and emergency procedures were excluded. The first 25 procedures performed with the Vessel Sealer per center were excluded to correct for effect of the initial robotic learning curve [13]. Results of robotic liver resections using the SynchroSeal were compared to those using the Vessel Sealer. The study is reported according to the Strengthening the Reporting of Observational Studies in Epidemiology (STROBE) Statement [14].

### Data collection

Procedures performed with the SynchroSeal and Vessel Sealer were identified from the Amsterdam UMC MILS Registry and a local MILS database of AZ Groeninge. The databases are prospectively maintained and contain data on patient, disease and procedure characteristics along with peri- and postoperative outcomes. The following patient characteristics were collected: age at time of operation, sex, body mass index (BMI), American Society of Anesthesiologists (ASA) score, use of neoadjuvant chemotherapy, presence of Cirrhosis, previous abdominal surgery and previous liver surgery. Disease characteristics of interest were histopathological diagnosis, largest tumour size, number of lesions on CT and presence of bilobar disease. Procedure characteristics included year of operation, type of hepatectomy (minor, technically major, anatomically major), extent of resection (wedge, segmentectomy, bi-segmentectomy, tri-segmentectomy, (extended) left/right hemi-hepatectomy), concurrent ablation, and concurrent operation (excluding cholecystectomy and lymphadenectomy). Intraoperative outcomes were operation duration, estimated intraoperative blood loss, major blood loss, use and duration of the Pringle manoeuvre, rate of conversion to open surgery and use of intraoperative transfusion. Postoperative outcomes were postoperative length of hospital-stay, Intensive Care Unit (ICU) admission, morbidity, severe morbidity, bile leak, readmission, reintervention, reoperation, 30-day or in-hospital mortality, resection margin status and textbook outcome in liver surgery (TOLS). Outcomes were reported with a follow-up of thirty days.

### Definitions

Liver segments are classified according to the Couinaud classification [15]. Patients were classified into three groups, minor, technically major and anatomically major, according to the extent and location of the resection performed. Minor resections were defined as resections of segments 2, 3, 4b, 5 or 6, involving less than three adjacent liver segments. Technically major resections are resections of segments 1, 4a, 7 or 8 involving less than three adjacent liver segments. Anatomically major resections refer to resections involving three or more adjacent liver segments. Intraoperative incidents were graded according to the Oslo classification [16]. The presence of cirrhosis was defined based on pathologic examination of the liver parenchyma. Operation duration was defined as the time in between the start of the skin incision until wound closure. Intraoperative blood loss was calculated by subtracting the volume of irrigation fluid from the volume of aspirated blood and blood absorbed by gauzes. Major blood loss was defined as intraoperative blood loss of 500 mL or more. Radical resection (R0) was characterized as histological evidence of tumour-free margins and at least 1 mm from the resection surface. Postoperative hospital length of stay is the number of nights the patient was admitted following the operation. Morbidity was graded according to the Clavien-Dindo classification [17]. With severe complications defined as Clavien-Dindo grade IIIa and above. Bile leaks were classified according to the standardized classification by the International Study Group of Liver Surgery (ISGLS) [18]. TOLS was defined using the same parameters as in the consensus definition, though limited to 30-days follow-up due to the available data [19]. To achieve TOLS all the following parameters must be met; absence of intraoperative incidents (grade ≥ 2), postoperative bile leakage (ISGLS grade B/C), postoperative liver failure (grade B/C), 30-day severe postoperative complications, 30-day readmission, 30-day or in-hospital mortality and the presence of R0 resection margin. Additionally, the extended definition of TOLS was used (TOLS +), this involves all the previously listed parameters as well as the absence of prolonged postoperative length of stay. Prolonged length of stay was defined as > 3 days, > 5 days and > 10 days for minor, technically major and anatomically major resections.

### Surgical technique

**Fig. 1:**
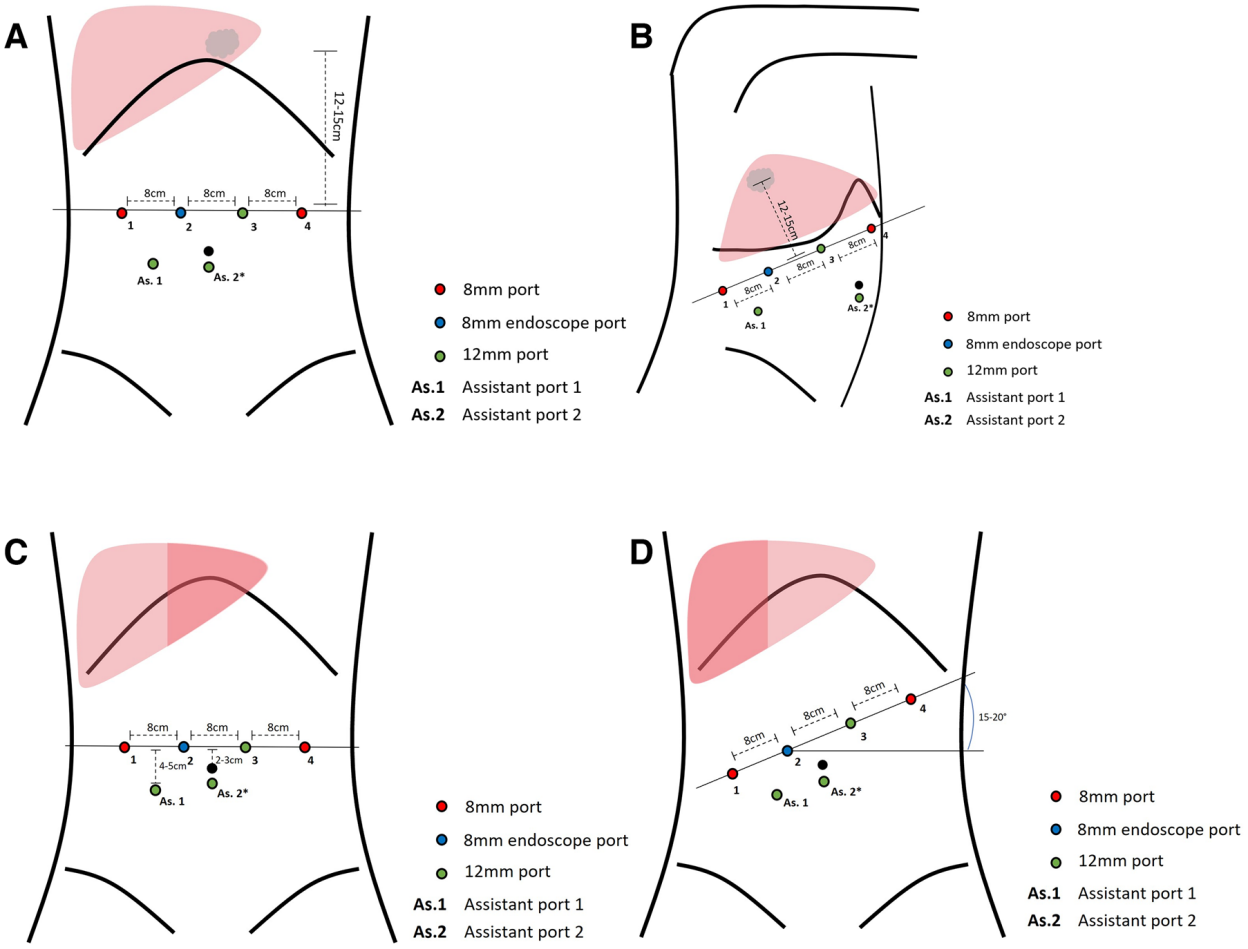
Port placement per hepatectomy type. Partial anterolateral resection (**A**), partial posterosuperior resection (**B**), left hemihepatectomy (**C**), and right hemihepatectomy (**D**). *Planned site of specimen extraction, location may differ depending on method of extraction. The second assistant port is optional and is primarily used in larger resections.

All surgeons had completed their learning curve for robotic liver surgery (> 25 procedures) prior to the introduction of the SynchroSeal at their respective centres [13]. Whereas the Vessel Sealer device was introduced at the time of implementation of robotic liver surgery at both centres. The operating teams consisted of at least one console surgeon and one bedside surgeon (surgeon, resident or fellow). The surgical technique was not altered throughout the study period. All procedures were completed using the DaVinci Xi platform (Intuitive Surgical, Inc., Sunnyvale, CA). Trocar placement was dependent on resection size and location as depicted in Fig. 1. Intraoperative ultrasound and indocyanine green (ICG) fluorescence imaging were used to demarcate the lesion(s), identify its relation to relevant anatomical structures and assess resection margins intraoperatively. The Pringle manoeuvre was applied when deemed necessary in periods of up to 20 min followed by a 5-min reperfusion period and repeated maximally three times. When applied, the Pringle manoeuvre was performed intracorporeally using the Huang Loop technique [20]. The energy device (SynchroSeal or Vessel Sealer) was placed in the third robotic arm and applied during at least part of the parenchymal transection. Vessels were ligated using titanium clips, Hem-O-Lok clips (Teleflex Inc., Morrisville, NC, USA) or staplers depending on size and anatomy. Use of haemostatic sealants and abdominal drains was left at the discretion of the operating surgeon. Autologous blood transfusion was only used at one of the institutions and only utilized for anatomically major resections or resections with a large transection plane. To ensure a hypovolemic state, the autologous blood was reinfused after parenchymal transection. Enhanced Recovery After Surgery (ERAS) protocols were in effect at both institutions.

### Ethics and privacy

Ethical approval for the use of patient data for the study was obtained from the medical ethics committee of the Amsterdam UMC (2023.0279) and AZ Groeninge Hospital (AZGS2023028) who waived the need for informed consent. The study was performed in accordance with the ethical principles outlined in the Declaration of Helsinki.

### Statistical analysis

Continuous variables are expressed as means with standard deviation (SD) if normally distributed or as medians with interquartile range (IQR) in the case of non-parametric data. Categorical data is displayed as absolute numbers with percentages. Comparisons of continuous data were made using the independent samples t-test if normally distributed and Mann–Whitney U when non-normally distributed. Comparisons of categorical data were made using the chi-squared test or Fisher’s exact test when appropriate. Propensity score matching was employed to mitigate selection bias, utilizing a 1:1 nearest neighbour matching approach with a caliper width set at 0.1. The groups were matched for the following variables: age, BMI, sex, ASA grade, presence of cirrhosis, neoadjuvant chemo, history of extrahepatic abdominal surgery, history of liver surgery, pathological diagnosis, largest lesion size, number of lesions, presence of bilobar disease, type of resection, resection difficulty (minor, technically major, anatomically major) concurrent ablation and concurrent operation. Missing data regarding ASA grade (*n* = 4, 1.3%) and size of largest lesion (*n* = 13; 4.3%) were imputed by means of single imputation. Paired t-test and Wilcoxon signed-rank test were utilized to compare the matched groups for continuous and categorical data respectively. All analyses were performed using IBM SPSS Statistics for Windows version 26.0 (SPSS V26.0, Inc., Chicago, IL, USA) and R for Windows version 4.3.2 (R Foundation for Statistical Computing, Vienna, Austria).

## Results

A total of 155 RLRs using the SynchroSeal (June 2021–March 2023) and 161 resections using the Vessel Sealer (February 2020–March 2023) were performed at the two institutions. During the study period only 7 procedures were performed without either of the energy devices and were therefore excluded from analysis. These resections involved small superficial tumours. Baseline characteristics are presented in Table 1.

**Table 1:** Baseline characteristics of robotic liver resection with the SynchroSeal and Vessel Sealer devices

	Unmatched				PSM 1:1			
	Vessel Sealer	SynchroSeal	*p*-value	SMD	Vessel Sealer	SynchroSeal	*p*-value	SMD
	*n* = 145	*n* = 155			*n* = 94	*n* = 94		
*Year of Surgery (%)*			** < 0.001**	0.879			** < 0.001**	0.905
2020	33 (22.8)	0 (0.0)			20 (21.3)	0 (0.0)		
2021	63 (43.4)	59 (38.1)			43 (45.7)	33 (35.1)		
2022	33 (22.8)	74 (47.7)			21 (22.3)	47 (50.0)		
2023	16 (11.0)	22 (14.2)			10 (10.6)	14 (14.9)		
*Age, years (median [IQR])*	63 [56—73]	66 [57—74]	0.413	0.130	65 [56—74]	65 [58—72]	0.997	0.053
Male sex (%)	82 (56.6)	91 (58.7)	0.705	0.044	59 (62.8)	56 (59.6)	0.779	0.066
BMI (median [IQR])	26.5 [23.8—30.1]	26.2 [23.3—29.9]	0.705	0.033	26.6 [23.8—30.1]	26.1 [23.3—30.0]	0.889	0.025
ASA score ≥ 3 (%)	41 (29.1)	56 (36.1)	0.197	0.151	30 (31.9)	29 (30.9)	1.000	0.023
Presence of cirrhosis (%)	15 (10.3)	24 (15.5)	0.186	0.154	13 (13.8)	14 (14.9)	1.000	0.030
*Pathological Diagnosis (%)*			**0.018**	0.467			0.996	0.202
CRLM	67 (46.2)	75 (48.4)			46 (48.9)	48 (51.1)		
HCC	25 (17.2)	26 (16.8)			19 (20.2)	16 (17.0)		
Cholangiocarcinoma	10 (6.9)	7 (4.5)			3 (3.2)	5 (5.3)		
Gallbladder Carcinoma	4 (2.8)	6 (3.9)			3 (3.2)	3 (3.2)		
Non-CRLM	3 (2.1)	17 (11.0)			3 (3.2)	3 (3.2)		
Other malignancy	6 (4.1)	1 (0.6)			0 (0.0)	1 (1.1)		
Benign	30 (20.7)	23 (14.8)			20 (21.3)	18 (19.1)		
Malignancy (%)	115 (79.3)	132 (85.2)	0.184	0.154	74 (78.7)	76 (80.9)	0.845	0.053
Neoadjuvant chemo (%)	40 (27.6)	35 (22.6)	0.317	0.116	20 (21.3)	20 (21.3)	1.000	< 0.001
History of extrahepatic abdominal surgery (%)	90 (62.1)	86 (55.5)	0.247	0.134	55 (58.5)	55 (58.5)	1.000	< 0.001
History of liver surgery (%)	15 (10.3)	32 (20.6)	**0.014**	0.288	13 (13.8)	11 (11.7)	0.814	0.064
Largest lesion size, mm (median IQR])	30 [20—44]	24 [15—37]	**0.022**	0.199	29 [16—40]	27 [15—40]	0.720	0.035
Number of lesions (median [IQR])	1 [1, 2]	1 [1, 2]	0.539	0.046	1 [1, 2]	1 [1, 2]	0.868	0.020
Presence of bilobar disease (%)	29 (20.0)	27 (17.4)	0.566	0.066	17 (18.1)	15 (16.0)	0.838	0.057
*Type of resection (%)*			0.146	0.228			0.918	0.076
Minor	67 (46.2)	68 (43.9)			43 (45.7)	41 (43.6)		
Technically major	58 (40.0)	75 (48.4)			43 (45.7)	43 (45.7)		
Anatomically major	20 (13.8)	12 (7.7)			8 (8.5)	10 (10.6)		
*Extent of resection (%)*			**0.033**	0.410			0.571	0.209
Wedge/non-anatomical	56 (38.6)	67 (43.2)			41 (43.6)	36 (38.3)		
Segmentectomy	35 (24.1)	55 (35.5)			25 (26.6)	30 (31.9)		
Bisegmentectomy	34 (23.4)	21 (13.5)			20 (21.3)	18 (19.1)		
Trisegmentectomy	3 (2.1)	1 (0.6)			0 (0.0)	1 (1.1)		
(Extended) left hemihepatectomy	12 (8.3)	5 (3.2)			5 (5.3)	5 (5.3)		
(Extended) right hemihepatectomy	5 (3.4)	6 (3.9)			3 (3.2)	4 (4.3)		
Concurrent ablation (%)	15 (10.3)	31 (20.0)	**0.020**	0.272	14 (14.9)	17 (18.1)	0.689	0.086
Concurrent operation (%)	24 (16.6)	8 (5.2)	**0.001**	0.372	5 (5.3)	7 (7.4)	0.752	0.087

*PSM* Propensity score matched, *SMD* Standardized mean difference, *BMI* Body Mass Index, *ASA* American Society of Anaesthesiologists, *CRLM* colorectal liver metastasis

### Perioperative outcomes of RLR with the SynchroSeal

Only one procedure (0.6%) with the SynchroSeal device had to be converted to open surgery. In this patient, the combination of a fibrotic and easily-bleeding liver with hemodynamic instability caused by the Pringle manoeuvre, necessitated conversion. In 52.9% of patients a Pringle manoeuvre was performed. When applied, total Pringle duration was median 30 min (IQR 20–40 min). Operating time was median 150 min (IQR 120–198 min). Eleven patients (7.1%) in the SynchroSeal group had a grade 1 intraoperative incident. One patient, being the converted procedure, had a grade 2 complication. Grade 1 incidents consisted of 4 bile leaks solved by suturing or ligation with clips, 4 bleedings solved by suturing or stapling, a serosal injury which was sutured, and an air embolus with a refractory oxygen saturation drop.

Following surgery, patients were admitted for a median of 3 days (IQR 2–4). 30-days postoperatively 19% of patients had experienced a postoperative complication, including the 11% which had experienced a severe (Clavien-Dindo ≥ 3A) complication. Of these patients, three required admission to the ICU for a median of 8 days (IQR 2–8). Bile leaks were experienced by 9 patients (5.8%) in the SynchroSeal group, of which 8 required a reintervention. No patients experienced postoperative bleeding from the liver resection surface.

Two patients (1.3%) had died at 30-days follow-up, one cirrhotic patient due to liver failure and the other as a result of a complicated postoperative course with a lung embolus and bleeding from the inferior epigastric artery requiring a reoperation during which iatrogenic damage of the bladder occurred. Later, the patient died of a haemorrhagic shock stemming from a bleeding at the site of the nephrostomy catheter.

Textbook outcome in liver surgery was achieved by 71.6% of patients following resection with the SynchroSeal. When prolonged length of stay was added to the definition (TOLS +) only 64.5% of patients achieved textbook outcome. The main reason for not achieving TOLS was an incomplete oncological resection (≥ R1). Histopathological examination found 88% of the patients with a malignant diagnosis had disease-free resection margins (R0).

### SynchroSeal versus Vessel Sealer

Patient, disease and procedure characteristics are presented in Table 1. Procedures with the SynchroSeal were performed more often in the latter years as compared with the Vessel Sealer (*p* < 0.001). The indication for resection differed significantly for the two devices (*p* = 0.018), though the proportion of malignant lesions were comparable between the groups (85.2% vs 79.3%, *p* = 0.184). The SynchroSeal group had a larger proportion of patients with a history of liver surgery (20.6% vs 10.3%, *p* = 0.014), a smaller largest lesion size (median 30 mm (IQR 20–44) vs 24 mm (IQR 15–37), *p* = 0.022), more often underwent concurrent ablation procedures (20% vs 10.3%, *p* = 0.020) though underwent concurrent operations less often (16.6% vs 5.2%, *p* = 0.001). Additionally, resection extent varied between the groups (*p* = 0.033). After PSM, the groups were well-balanced regarding baseline characteristics and contained 94 patients per group. Year of surgery was not taken into account during matching and remained significantly different (*p* < 0.001).

Perioperative outcomes of RLR with the SynchroSeal and Vessel Sealer were largely comparable in the matched groups. (Table 2) Liver resections with the SynchroSeal were accompanied by significantly less blood loss (48 mL [IQR 10–143] vs 95 mL [IQR 30 -200], *p* = 0.032) though had similar rates of major blood loss (6.4% vs 3.3%, *p* = 0.505). Other intraoperative outcomes including operative time, Pringle use, Pringle duration, intraoperative transfusions, and intraoperative incidents were comparable between the devices. Moreover, postoperative outcomes showed no significant differences. TOLS rates were similar for both devices (TOLS: 78.7% vs 74.5%, *p* = 0.651; TOLS + : 68.1% vs 63.8%, *p* = 0.683).

**Table 2:** Perioperative outcomes of robotic liver resection with the SynchroSeal and Vessel Sealer devices

	Unmatched			PSM 1:1		
	Vessel Sealer	SynchroSeal	*p*-value	Vessel Sealer	SynchroSeal	*p*-value
*n*	*n* = 145	*n* = 155		*n* = 94	*n* = 94	
Intraoperative blood loss, mL (median [IQR])	50 [20—150]	40 [10—100]	**0.002**	95 [30—200]	48 [10—143]	**0.032**
Major blood loss (> 500 mL) (%)	7 (4.9)	7 (4.5)	0.887	3 (3.3)	6 (6.4)	0.505
Pringle manoeuvre (%)	71 (49.0)	82 (52.9)	0.495	41 (43.6)	52 (55.3)	0.145
Pringle duration when applied (median [IQR])	29 [20—40]	30 [20—40]	0.685	31 [23—47]	35 [20—45]	0.862
Operative time (median [IQR])	165 [130—228]	150 [120—198]	0.059	181 [134—239]	154 [120—210]	0.160
Intraoperative transfusion (%)	1 (0.7)	2 (1.3)	0.610	0 (0.0)	2 (2.1)	0.480
*Intraoperative Incidents, Oslo classification (%)*			0.511			0.988
0	132 (91.0)	143 (92.3)		86 (91.5)	84 (89.4)	
1	9 (6.2)	11 (7.1)		6 (6.4)	9 (9.6)	
2	3 (2.1)	1 (0.6)		2 (2.1)	1 (1.1)	
3	1 (0.7)	0 (0.0)		0 (0.0)	0 (0.0)	
Conversion (%)	5 (3.4)	1 (0.6)	0.083	2 (2.1)	1 (1.1)	1.000
Length of hospital stay, days (median [IQR])	3 [2–4]	3 [2–4]	0.112	3 [2–4]	3 [2–4]	0.446
ICU admission (%)	6 (4.1)	3 (1.9)	0.264	5 (5.3)	1 (1.1)	0.221
Overall morbidity (%)	27 (18.6)	30 (19.4)	0.871	18 (19.1)	18 (19.1)	1.000
*Highest complication grade, Clavien-Dindo (%)*			0.956			1.000
1	7 (4.8)	6 (3.9)		76 (80.9)	76 (80.9)	
2	7 (4.8)	7 (4.5)		3 (3.2)	6 (6.4)	
3a	7 (4.8)	7 (4.5)		6 (6.4)	4 (4.3)	
3b	3 (2.1)	7 (4.5)		2 (2.1)	4 (4.3)	
4a	1 (0.7)	1 (0.6)		1 (1.1)	0 (0.0)	
4b	0 (0.0)	0 (0.0)		0 (0.0)	0 (0.0)	
5	2 (1.4)	2 (1.3)		1 (1.1)	1 (1.1)	
Severe complications (%)	13 (9.0)	17 (11.0)	0.563	10 (10.6)	9 (9.6)	1.000
Bile leak (%)	4 (2.8)	9 (5.8)	0.195	3 (3.2)	6 (6.4)	0.505
Readmission (%)	6 (4.2)	11 (7.1)	0.274	5 (5.3)	5 (5.3)	1.000
Reintervention (%)	12 (8.3)	16 (10.3)	0.543	10 (10.6)	8 (8.5)	0.814
Reoperation (%)	2 (1.4)	2 (1.3)	0.946	2 (2.1)	0 (0.0)	0.480
30-day or in-hospital mortality (%)	2 (1.4)	2 (1.3)	0.946	1 (1.1)	1 (1.1)	1.000
Resection margin, R0*(%)	94 (81.7)	116 (88.0)	0.469	60 (81.1)	66 (86.8)	0.275
TOLS (%)	110 (75.9)	111 (71.6)	0.404	70 (74.5)	74 (78.7)	0.651
TOLS + (%)	92 (63.4)	100 (64.5)	0.847	60 (63.8)	64 (68.1)	0.683

*PSM* Propensity score matched, *ICU* Intensive Care Unit, *TOLS* Textbook Outcomes in Liver Surgery

*In malignant cases

## Discussion

We present the results of RLR performed using the SynchroSeal in a heterogenous group of patients in two hospitals. Our findings suggest that the SynchroSeal device is a safe and effective tool for robotic parenchymal liver transection in experienced hands. Intraoperative as well as postoperative outcomes were favourable and support the safety and efficiency of the device. Only one patient required a conversion and an acceptable number of patients experienced severe postoperative complications 17 (11%). A small proportion of patients (5.8%) experienced a bile leak and there were no postoperative bleedings from the liver surface, speaking to the adequate sealing qualities of the SynchroSeal. Moreover, when compared with the more widely used, previous generation Vessel Sealer, the SynchroSeal device was associated with less intraoperative blood loss, however major blood loss was comparable. Other perioperative outcomes were similar between the two devices.

In recent years, there has been a remarkable surge in the volume of robotic-assisted surgery procedures [21, 22]. Although the implementation of robotics in liver surgery lagged behind other surgical fields, a similar booming trend is now being observed in hepatic surgery [23, 24]. However, hepatobiliary surgeons seem to remain unsatisfied with the available equipment, specifically for parenchymal transection of the liver [25]. To support the ongoing development of robotic liver surgery we must critically evaluate and reflect on the outcomes of new surgical devices introduced into the market before widespread implementation takes place [26]. In light of this, we presented our experience with a novel robotic energy device.

Our experience is that the SynchroSeal device is valuable for precise parenchymal transection. The slim instrument jaws and wrist-like function allow for delicate dissection of intraparenchymal structures such as bile ducts and vasculature prior to ligation and division. The SynchroSeal could effectively transect liver tissue and seal minor vasculature and bile ducts. However, it is not suitable for sealing larger structures, so this must be complemented with other tools such as clips, staplers and sutures to ensure adequate hemo- and biliostasis. At both centres, surgeons had previously employed the Vessel Sealer energy device for robotic parenchymal transection, however, were discontent with its bulky nature. Following implementation of the SynchroSeal, it has become the instrument of choice for conducting robotic liver resections at both institutions. Some surgeons have criticized the SynchroSeal for the lack of a (physical) cutting function, we did not miss this feature during parenchymal transection, as both sealing (blue pedal) and cutting (yellow pedal) can be performed using bipolar energy. This seems to be, as with many surgical instruments, a matter of experience. Additionally, the SynchroSeal and Vessel Sealer are offered for equivalent prices meaning economic considerations should not play a role in the choice between these two instruments.

Comparison of outcomes of parenchymal transection performed with the SynchroSeal versus Vessel Sealer revealed that the SynchroSeal was associated with less intraoperative blood loss. However, the clinical relevance of this finding is minimal (< 50 ml). Regarding other perioperative outcomes, the devices offered similar outcomes. It must be acknowledged that comparison of these devices is subject to bias due to the later implementation of the SynchroSeal at both centres and results should be interpreted accordingly. The finding of statistically significant lower blood loss in resections performed with the SynchroSeal can more likely be attributed to the learning curve rather than sealing capabilities of the device. On a favourable note, these data suggest that exceptional outcomes can be achieved with both devices and surgeons using the Vessel Sealer can smoothly transition to the SynchroSeal without compromising outcomes.

Although our series had technically more complex cases, a Dutch nationwide analysis found similar outcomes following robotic liver surgery, including length of hospital stay, rate of (severe) complications, mortality and R0 resection [23]. The resections in the Dutch series were primarily performed using the Vessel Sealer. Intraoperative outcomes including blood loss (150 mL vs. 40 mL) and conversion rate (6.3% vs. 0.6%) were substantially more favourable in our cohort. Again, it is unclear whether this can be solely attributed to the SynchroSeal device since the previous studies encompassed cases from the initial phase of the learning curve, whereas ours did not.

The proportion of patients in the SynchroSeal group achieving TOLS was 71.6%, generally corresponding with published results. Gorgëc et al. reported a TOLS rate of 69%, while de Graaff et al. found TOLS rates ranging from 51% for biliary cancers to 80% for colorectal liver metastasis [27, 28]. Additionally, Tsilimigras et al. found a TOLS rate of 62.0% and 62.3% in primary liver cancers and hepatocellular carcinoma, respectively [29, 30]. It is important to note that the use of diverse definitions of textbook outcome across literature limit the direct comparisons that can be made regarding this composite outcome. The aforementioned studies did not incorporate intraoperative incidents in their definition of TOLS, while the present study did. R0 resection was the primary limitation for achieving TOLS in the present study. Of note, is that we did not distinguish vascular R1 resections from R1 resections and results should therefore be interpreted accordingly. R1 vascular resection are not necessarily associated with poorer oncological outcomes [31, 32]. The proportion of patients with R0 resections (SynchroSeal 88%, Vessel Sealer 82%) in the current cohort, mirrors rates documented in population-based studies [23, 33, 34].

The outcomes of robotic parenchymal transection with the SynchroSeal are promising, however, they must be considered in light of several limitations. Firstly, patients were operated in high volume centres by surgeons with extensive experience with robotic hepatectomy. The surgeons’ extensive experience in this cohort is reflected by the low conversion rate. Additionally, the proportion of anatomically major resections (8%) was relatively low. This can be partly explained by the relatively high rate of combined resection and ablations (20%) potentially resulting in more parenchymal sparing resections [35]. It should therefore be noted that these outcomes cannot be generalized for all centres performing hepatobiliary surgery. Results should be interpreted while keeping the high proportion of minor resections in mind. Based on our own experience with the device, we do not anticipate the SynchroSeal to present greater challenges to novice liver surgeons compared with other parenchymal transection tools. Secondly, although PSM was applied to limit selection bias, this method only corrects for known confounders. Additionally, although the first 25 procedures with the Vessel Sealer were excluded it can be expected that surgeons’ skills will continuously develop. Therefore, a learning curve effect could not be completely mitigated owing to the later implementation of the SynchroSeal. It remains to be elucidated whether the SynchroSeal has improved performance compared to other conventional instrumentation used for robotic hepatectomy. Thirdly, the use of various ‘best practice’ techniques in liver surgery such as the use of the Pringle manoeuvre and devices such as vascular staplers, bipolar energy and haemostatic sealants undoubtably also contributed to the excellent results achieved, that cannot all be attributed to the SynchroSeal device. Fourthly, regrettably, the degree to which the energy devices were utilized during the parenchymal transection phase was not documented as well as the parenchymal transection time. We therefore only know that a certain device was utilized, yet not for how long. Such data would have allowed better insight into the efficiency and role of the SynchroSeal in this critical phase of the operation. Fifthly, due to a short follow-up period no conclusions can be drawn regarding oncological outcomes.

To the best of our knowledge, this is the first cohort reporting on the use of the SynchroSeal device for parenchymal liver transections. The adequate outcomes achieved in our cohort, support the continued implementation of this device in practice.

## Conclusion

The utilization of the SynchroSeal device for parenchymal transection in RLRs has been found to be both safe and effective.
